# Diabetes mellitus and the risk of total knee replacement among Chinese in Singapore, the Singapore Chinese Health Study

**DOI:** 10.1038/srep40671

**Published:** 2017-01-13

**Authors:** Ying-Ying Leung, John Carson Allen, Li-Wei Ang, Jian-Min Yuan, Woon-Puay Koh

**Affiliations:** 1 Duke-NUS Medical School, Singapore; 2 Department of Rheumatology and Immunology, Singapore General Hospital, Singapore; 3 Epidemiology & Disease Control Division, Ministry of Health, Singapore; 4 Division of Cancer Control and Population Sciences, University of Pittsburgh Cancer Institute, Pittsburgh, Pennsylvania, USA; 5 Department of Epidemiology, Graduate School of Public Health, University of Pittsburgh, Pittsburgh, Pennsylvania, USA; 6 Saw Swee Hock School of Public Health, National University of Singapore, Singapore.

## Abstract

Association between diabetes mellitus (diabetes) and risk of knee osteoarthritis (KOA) is confounded by high body mass index (BMI), a strong risk factor for both conditions. We evaluated the association between diabetes and incidence of total knee replacement (TKR) due to severe KOA in the Singapore Chinese Health Study, a prospective cohort of 63,257 Chinese men and women, aged 45–74 years at recruitment in 1993–1998, and re-interviewed in 1999–2004. Height, weight, lifestyle factors and history of diabetes were obtained through in-person interviews at recruitment and re-interview. Incident cases of TKR were identified via record linkage with nationwide hospital discharge database. Subjects with/without prevalent diabetes had comparable BMI (24.0 kg/m^2^ versus 23.0 kg/m^2^). After an average of 14-years, 1,973 subjects had TKR attributable to KOA. Compared to subjects without diabetes, hazard ratio (HR) of TKR for subjects with diabetes was 0.63 [95% confidence interval (CI), 0.52–0.75] after controlling for BMI and other risk factors. An inverse association was also observed between incident diabetes at re-interview and subsequent risk of TKR (HR = 0.74; 95% CI = 0.58–0.94). The inverse diabetes-TKR risk association was similar by gender and across three categories of BMI. Our study does not support diabetes as a risk factor of KOA.

Knee osteoarthritis (KOA) is among the leading causes of disability, imposing a heavy burden on individuals and population health globally^1^,^2^. Apart from age, KOA is often attributed to the biomechanical stress caused by weight-loading from obesity^3^,^4^. In a previous study, we have demonstrated a positive, log-linear relationship between body mass index (BMI) and risk of total knee replacement (TKR) for severe KOA over a wide range of BMI, beginning with a level as low as 16 kg/m^2^, in a population-based cohort of Singapore Chinese^5^. Our study, together with many other studies^3^,^4^,^6^,^7^, has established increased BMI or as a strong risk factor for severe KOA.

Recent data from epidemiologic studies suggest that diabetes mellitus (diabetes) and/or hyperglycaemia may also be risk factors for severe KOA^8^. Obesity also is a well-established strong risk factor for diabetes^9^,^10^. Therefore, it is not unexpected that in most of these studies conducted among Western populations, subjects with diabetes or hyperglycemia had much higher BMI compared to those without these conditions^11^,^12^,^13^. For example, in a cohort study in Italy, subjects with diabetes had a mean BMI of 27.0 kg/m^2^ compared to 24.8 kg/m^2^ in subjects without diabetes^12^. Hence, it is challenging to eliminate residual confounding effect of obesity on the association between diabetes and KOA risk. Even in experimental animal studies, it is difficult to separate the biomechanical effects of obesity from the effects of diabetes^14^, since the animals with induced diabetes either from genetic manipulation or high fat diet were also more obese than the control animals without induced diabetes^15^. Therefore, epidemiological studies in populations with low BMI are needed to minimize the confounding effect of obesity on the diabetes-KOA risk association.

Asians, in general, are leaner than Caucasians^16^. Furthermore, in Asians, diabetes is characterized by onset at lower BMI, and subjects with diabetes have only marginally elevated BMI relative to their counterparts without diabetes^17^, compared to the greater disparity in BMI between subjects with and without diabetes in Western population studies^11^,^12^,^13^. In a prospective cohort study in Japan, high glycated haemoglobin (HbA1c) was not significantly associated with either onset or progression of radiographic KOA after adjustment for BMI^18^. In this study, we used data from the Singapore Chinese Health Study (SCHS), a prospective cohort of middle-aged or older Chinese men and women with an average baseline BMI of 23 kg/m^2^ compared to 24 kg/m^2^ for subjects with diabetes, to assess the effect of diabetes on risk of TKR due to KOA independent of obesity.

## Research Design and Methods

### Study population

The SCHS is a prospective cohort study that enrolled 63,257 Chinese (27,959 men and 35,298 women) at ages 45–74 years between 1993 and 1998 in Singapore^19^. Subjects were recruited from public housing estates, where 86% of Singapore’s population lived at the time of recruitment. Study subjects were restricted to the two major dialect groups in Singapore: Hokkien and Cantonese, originating from the Fujian and Guangdong Provinces in Southern China, respectively. This study was approved by the Institutional Review Boards at the National University of Singapore. All methods were carried out in accordance with the Declaration of Helsinki, and all subjects have signed informed consent prior to participation.

### Exposure assessment

At recruitment, the assessment was conducted via in-person interview using a structured questionnaire. Information was obtained on education level, cigarette smoking, alcohol consumption, habitual physical activity, sleep hours and habitual dietary intake via a validated 165-item food frequency questionnaire. Medical history of diabetes, coronary heart disease and stroke was enquired by “Yes/No” to the following question, “Please tell me if you have been told by a doctor to have this condition”; and “If yes, tell me the age at which you were first diagnosed with the condition”. The robustness and accuracy (98.9%) of the self-reported diabetes data in this cohort was validated in a separate study analysing 1,651 cohort subjects^20^.

Body weight and height at baseline were self-reported during the interview, and BMI was calculated as weight in kilograms divided by height in meters squared (kg/m^2^). A total of 10,349 cohort subjects (16%) did not report either weight and/or height, and BMI was calculated using imputed weight and/or height obtained from the linear regression equation: weight = y-intercept + gradient × height, where values for the y-intercept and gradient were derived from gender-specific weight-height regression lines obtained from all subjects with known heights and weights. This method of imputed BMI was reported in detail previously^21^.

A follow-up interview of the SCHS cohort was conducted between 1999 and 2004, and 83% of the original cohort was re-interviewed. Information on self-reported medical history (diabetes, coronary heart disease, and stroke), weight and height was obtained again using the same questions asked during the baseline interview at recruitment.

### Identification of incident cases of TKR for severe KOA

We identified cases of TKR for severe KOA in this cohort via record linkage with the nationwide MediClaim System hospital discharge database through 31 December 2011. The system has been in use in Singapore since 1990 and captures surgical procedures and up to 3 discharge diagnoses per patient for hospitalizations in public and private hospitals based on the ninth revision of the International Statistical Classification of Diseases and Related Health Problems (ICD-9). We included only subjects who underwent TKR for severe KOA (ICD-9 code 715) as cases for the first time, and excluded those who underwent TKR for diagnoses such as septic arthritis, osteomyelitis, villonodular synovitis, rheumatoid arthritis, psoriatic arthritis, ankylosing spondylitis, and other inflammatory arthritis, or secondary causes of KOA such as avascular or aseptic necrosis of joint, meniscus or ligament injuries, and other congenital or acquired deformities of the knee (n = 89). A total of 128 prevalent cases of TKR which occurred prior to subject recruitment into the cohort were excluded from analysis^22^. Deaths were identified through record linkage with the Singapore Registry of Births and Deaths. As of December 2011, only 47 subjects were known to be lost to follow-up due to migration out of Singapore or for other reasons, suggesting the ascertainment of vital status for cohort subjects is virtually complete.

### Statistical analysis

We used the chi-square test for categorical variables or the Student’s t-test for continuous variables to examine differences in baseline characteristics between subjects with and without diabetes. We used Cox proportional hazards regression to investigate the risk of TKR in association with self-reported physician-diagnosed diabetes status (yes/no). Three hierarchical Cox regression models were used for data analysis for all subjects and for men and women separately. Model 1 included the following covariates: age at recruitment (years), year of recruitment (1993–1995, 1995–1998), dialect group (Hokkien, Cantonese), and level of education (no formal education, primary school, secondary school or higher). Model 2 included all covariates in Model 1 and BMI at baseline. Model 3 included additional variables as follows: smoking status (never, former, or current), self-reported histories of physician-diagnosed coronary artery disease and stroke, as well as number of hours per week spent in moderate physical activity, vigorous work, and strenuous sports. Hazard ratios (HRs) with 95% confidence intervals (CIs) were derived from Cox regression models to assess the strength of the association between diabetes and risk of TKR with adjustment for different sets of covariates. Heterogeneity of the diabetes-TKR risk association across different major confounding factors was tested using a product term between diabetes status and the factor of interest in the Cox model. We also performed sensitivity analysis by including only participants who were able to report both weight and height for computation of BMI.

Among the 63,129 subjects, 52,221 (83%) participated in follow-up interview between 1999 and 2004. We excluded 4,022 subjects with prevalent diabetes at recruitment and further 330 cases with TKR between recruitment and follow-up interview, and performed an analysis among the remaining 47,869 subjects to investigate the association between incident diabetes reported at follow-up and subsequent risk of TKR.

In order to further control for BMI, we performed subgroup analyses for diabetes and risk of TKR within each narrow BMI categories based on the World Health Organization (WHO) public health action cut-offs for Asians that better defined cardiovascular risk^16^: <23, 23 to <27.5, and ≥27.5 kg/m^2^. HR estimates for diabetes status within the BMI categories were further adjusted for BMI (in continuous value) and all other potential lifestyle and comorbidity confounders as covariates.

All statistical analysis was conducted using SAS Version 9.2 (SAS Institute, Inc., Cary, North Carolina). All reported *p* values were two-sided, and *p* < 0.05 was considered statistically significant.

## Results

After a mean (±SD) follow-up time of 14.5 ± 4.3 years, there were 1,973 incident cases of TKR due to severe KOA among the 63,129 subjects included in the analyses. Women accounted for 83.4% of TKR cases. The mean age at TKR was 67.8 ± 6.6 years. Table 1 summarizes the baseline characteristics of cohort participants with and without a history of diabetes at recruitment and follow-up interviews. Mean BMI among subjects with diabetes (24.0 ± 3.3 kg/m^2^) was about 1 kg/m^2^ higher than those without diabetes (23.0 ± 3.2 kg/m^2^). About 12.4% of subjects in the ≥25 kg/m^2^ BMI category reported a history of diabetes, followed by 10.0% in the 23–25 kg/m^2^ and 6.8% in the <23 kg/m^2^ categories. Subjects with diabetes were, on average, about 4 years older (60.1 ± 7.7) than subjects without diabetes (56.5 ± 8.0) at recruitment. Those with history of diabetes also had a lower level of education, and spent less time in physical activity compared to those without diabetes. There was a higher proportion of former smokers but a lower proportion of current smokers among those with diabetes. Subjects with diabetes also had higher prevalence of self-reported physician diagnosis of coronary heart disease (12.3 vs. 3.3%) and stroke (4.8 vs. 1.2%).

Among the 47,869 subjects with follow-up data included in the second analysis, 3,435 subjects reported to have incident diabetes diagnosed after recruitment. After a mean (±SD) follow-up time of 9.7 ± 2.5 years, there were 1,365 incident cases of TKR due to severe KOA. The comparisons of demographic characteristics between subjects with or without incident diabetes at follow-up were similar to those between subjects with and without prevalent diabetes at recruitment (Table 1).

Table 2 presents results of the hierarchical model regression analyses assessing risk of TKR associated with diabetes status at recruitment. The negative association between diabetes and TKR became stronger after adjusting for BMI (Model 2) and did not change substantially after additional adjustment for other lifestyle factors and comorbidity of coronary heart disease and stroke (Model 3). After adjusting for all other established and potential risk factors associated with severe KOA, including BMI, subjects with diabetes had a 37% reduction in risk of TKR (HR = 0.63; 95% CI = 0.52–0.75). Similar inverse associations were seen in men and women and there was no significant interaction between gender and diabetes status on risk of TKR (*p* for interaction = 0.99) (Table 2). We also conducted sensitivity analysis by excluding all subjects with imputed BMI in the analysis. Among the 52,780 subjects with reported weight and height for the computation of BMI, there were 1,649 cases of TKR. The results remained unchanged; subjects with diabetes had a 40% reduction in risk of TKR (HR = 0.60; 95% CI = 0.48–0.73).

When we confined our analysis to subjects without prevalent diabetes at recruitment, the inverse association between incident diabetes reported at follow-up and TKR remain unchanged after adjusting for BMI and all other established and potential risk factors associated with severe KOA (Table 3), and the risk estimates were again similar between men and women (*p* for interaction = 0.68).

To examine if the association between diabetes and risk of TKR may be different by BMI categories, we performed subgroup analysis stratified by three BMI categories. Hazard ratios of TKR for subjects with diabetes who had baseline BMI of <23, 23 to <27.5, and ≥27.5 kg/m^2^ were 0.56 (95% CI = 0.35–0.90), 0.66 (95% CI = 0.52–0.84) and 0.45 (95% CI = 0.30–0.67), respectively (Table 4). There was therefore no interaction between BMI categories and diabetes status on the risk of TKR (all *P’s* for interaction >0.35). Similarly, subjects with incident diabetes at follow-up had reduced risk of TKR, with HRs ranging from 0.66 to 0.82 in the subgroup analysis stratified by the three BMI categories (data not shown).

Finally, since patients with diabetes generally have higher mortality rate than those without diabetes, to exclude premature death as a potential competing outcome with TKR, we further excluded from the analyses subjects who died before the censored date of 31 December 2011, and this comprised 14,876 subjects (23.6% of cohort), including 173 cases of TKR (8.8% of TKR cases). The results remained essentially unchanged. Compared to subjects without diabetes, those with diabetes had a relative risk of 0.62 (95% CI = 0.50–0.76).

## Discussion

In this study, using subjects from the Singapore Chinese Health Study cohort, which is a relatively lean population with comparable BMI between subjects with or without diabetes, we found a statistically significant inverse association between diabetes and risk of TKR after controlling for BMI and other potential confounding factors.

A possible counter-argument to our finding is that subjects with diabetes are less likely to undergo TKR, especially among those with longer duration of diabetes since they are also more likely to have diabetes-related complications that could preclude surgery. Hence, we excluded subjects with prevalent diabetes at recruitment and studied the association between incident diabetes diagnosed after recruitment and the subsequent risk of TKR, and the inverse association was still observed. A survey among orthopaedic surgeons in New York City in the United States showed that although the decision against total knee replacement surgery made by the surgeon could be due to comorbidity, diabetes was not mentioned specifically as a factor^23^. Furthermore, in our study, the other comorbidity factors of stroke and coronary heart disease, which could affect fitness for surgery, were not associated with lower risk of TKR (data not shown). Another counter-argument against our finding is that since patients with diabetes generally have higher mortality rate than those without diabetes, the incidence of TKR in those with diabetes could be lower due to survival disadvantage. However, in this study, the inverse association between diabetes and TKR remained essentially unchanged when all subjects who died before the censored date were excluded from analysis. Thus the findings of an inverse diabetes-TKR risk association in this Chinese population with relatively low BMI are rigorous, and warrant further confirmation.

Several case-control^11^,^13^,^24^ cross-sectional^25^,^26^,^27^ and cohort^12^,^28^ studies showed positive associations of diabetes and/or hyperglycaemia with KOA. A common feature and the major limitation in these epidemiological studies was that subjects with diabetes or hyperglycemia had substantially higher BMI compared to those without the condition^11^,^12^,^13^,^24^,^25^,^26^, thus raising the possibility of residual confounding effect of BMI even after adjustment by statistical modelling. Furthermore, BMI was not adjusted for in some studies^25^,^26^,^28^. In a cohort study of 927 subjects in Italy^12^, BMI was considerably higher in those with diabetes than in those without diabetes (27.0 versus 24.8 kg/m^2^). When the authors adjusted for BMI as a continuous variable in their model, the risk estimate associating diabetes with risk of knee arthroplasty decreased markedly from 3.76 to 2.06, suggesting that the confounding effect of BMI was substantial, and residual confounding may remain from insufficient adjustment. In our study, the inverse association between diabetes and TKR was greatly strengthened when we adjusted for BMI, and this observation supports our speculation that other studies which have shown a positive association between diabetes and KOA may not have adequately adjusted for BMI since it is a strong negative confounding factor. Furthermore, our speculation is supported by a few studies with large sample sizes that did not find any significant association between diabetes, hyperglycemia or insulin resistance and risk of OA when BMI was included as a covariate in the model^18^,^29^,^30^,^31^,^32^,^33^,^34^. In a cross-sectional study in South Korea^32^, a number of components of metabolic syndrome (MetS), including waist circumference, hypertension, fasting hyperglycemia, hypertriglyceridemia and low HDL-cholesterol, were associated with radiographic KOA and knee pain. The association remained after adjusting for a surrogate of insulin resistance (homeostasis model assessment-estimated insulin resistance, HOMA-IR), but became non-significant after adjusting for weight or BMI, suggesting that the association between metabolic syndrome and radiographic KOA was largely explained by excessive weight but not by insulin resistance. In another prospective cohort study in Japan^18^, high glycated haemoglobin (HbA1c) was not significantly associated with either onset or progression of radiographic KOA after adjustment for BMI. Similarly, two recent large cohort studies in Sweden^33^ and Australia^34^ noted that the association between impaired fasting blood sugar >5.6 mmol/L or history of diabetes and TKR became non-significant, after adjustment for BMI was made.

To our best knowledge, our study is the first to report an inverse association between diabetes and risk of KOA, even after careful adjustment for BMI. These findings are contrary to other studies that have shown either a positive or null association between diabetes and KOA. The mechanisms underlying the potential effect of hyperglycemia or insulin resistance on KOA are not yet fully elucidated^14^. *In-vitro* studies have suggested that accumulation of advanced glycation end products (AGEs) in cartilage lead to matrix stiffness and sensitized cartilage to mechanical stress. Binding of AGEs to their ligands triggered activation of different signalling pathways leading to over-expression of pro-inflammatory mediators that lead to cartilage degradation. However, there have been no experimental studies that have assessed the level of AGE formation in cartilage among those with diabetes compared to those without^8^. On the contrary, hyperglycemia could depress the immune response in various ways, including impairment of neutrophil chemotaxis^35^, phagocytosis, opsonisation, and cell-mediated immunity, all of which may reduce local inflammatory in the joints. Tumor necrosis factor-alpha (TNFα) and interleukin (IL)-1β from lipopolysaccharide-stimulated macrophages are lower in diabetic mice compared to control mice^36^. In a small case-control study, subchondral sclerosis and osteophytes on knee radiography were less common in subjects with diabetes compared to controls matched by age, weight, and duration of OA symptoms, suggesting that a diabetes state may attenuate the chondro- and osteo-genesis required for osteophyte formation in OA^37^.

Moreover, drugs given to subjects with diabetes may have immunomodulation effects that slow down OA progression. Metformin, the most common diabetes medication, was shown to have immune-modulating effects on inflammatory arthritis in animal models via down-regulating IL-17-producing T (Th17) cells while activating and up-regulating regulatory T cells^38^,^39^.

The major strength of our study derives from the study being conducted in a relatively lean population where disparity BMI between subjects with and without diabetes is smaller than those reported in other published studies^11^,^12^,^13^,^25^,^26^, thus minimizing the potential for bias in estimating risk of TKR due to disparities in BMI. Other strengths include a population-based prospective cohort study design, a large number of identified TKR cases, long follow-up time, minimal recall bias in exposure since exposure information was collected prior to TKR, and complete case ascertainment of TKR for severe KOA through linkage with a comprehensive, nationwide hospital database. Possible limitations of this study include the use of TKR as a surrogate outcome for severe KOA, which may exclude subjects who had severe KOA but did not undergo surgery due to medical, financial or other reasons. However, Singapore is a small city-state where access to medical care is made affordable, and the comparatively high incidence of TKR in this cohort suggests adequate accessibility to TKR for severe KOA. Additionally, by using TKR as the outcome, we are unable to discern whether diabetes was associated with the onset or progression of KOA. Another limitation could be non-differential misclassification in using self-report of diabetes status, which could potentially result in underestimating the effect of diabetes on risk of TKR. Similarly, there could be non-differential bias in using self-reported body weight and height to compute BMI. However, self-reported body weight and height have been shown to be generally valid for epidemiologic studies across many populations^40^, and we have demonstrated that a strong linear, positive association existed between increasing BMI, computed from self-report weight and height, and increasing risk of TKR in this cohort in our previous study^5^. Lastly, we did not have information on blood sugar, diabetic control and treatment; therefore we cannot differentiate whether the observed inverse association between self-reported diabetes and risk of TKR is due to hyperglycemia per se or the result of treatment for hyperglycemia.

In conclusion, we have provided strong epidemiologic evidence that diabetes is not a risk factor for TKR due to severe KOA. On the contrary, we have shown a statistically significant inverse association between diabetes and risk of TKR. We recommend that future research includes epidemiologic studies that carefully address the possible residual confounding effects of BMI, as well as experimental studies to further delineate the biological effects of insulin resistance and/or hyperglycaemia in the pathophysiology of osteoarthritis.

## Additional Information

**How to cite this article**: Leung, Y.-Y. *et al*. Diabetes mellitus and the risk of total knee replacement among Chinese in Singapore, the Singapore Chinese Health Study. *Sci. Rep.* **7**, 40671; doi: 10.1038/srep40671 (2017).

**Publisher's note:** Springer Nature remains neutral with regard to jurisdictional claims in published maps and institutional affiliations.

## Figures and Tables

**Table 1:** Baseline demographic and lifestyle characteristics by history of diabetes at recruitment and follow-up.

	History of prevalent diabetes at recruitment	No history of diabetes at recruitment	History of incident diabetes at follow-up	No history of diabetes at follow-up
Number of subjects	5,671	57,458	3,435	44,434
Gender, n (%)				
Men	2,422 (42.7)	22,516 (44.4)	1,545 (45.0)	18,996 (42.8)
Women	3,249 (57.3)	31,942 (55.6)	1,890 (55.0)	25,438 (57.2)
Age at interview ± SD, years	60.1 ± 7.7	56.1 ± 8.0	74.0 (18.0)	69.5 (17.4)
BMI, mean ± SD	24.0 ± 3.3	23.0 ± 3.2	24.5 (3.7)	23.0 (3.5)
BMI categories, n (%)				
<23 kg/m^2^	2,056 (36.3)	28,289 (49.2)	1,189 (34.6)	22,886 (51.5)
23-> 25 kg/m^2^	1,909 (33.7)	17,151 (29.9)	1,597 (46.5)	17,228 (38.8)
>25 kg/m^2^	1,706 (30.1)	12,018 (20.9)	649 (18.9)	4,320 (9.7)
Level of education, n (%)				
No formal education	1,993 (35.1)	15,264 (26.6)	1,012 (29.5)	10,947 (24.6)
Primary	2,490 (43.9)	25,516 (44.4)	1,530 (44.5)	19,846 (44.7)
Secondary or above	1,188 (21.0)	16,678 (29.0)	893 (26.0)	13,641 (30.7)
Smoking status				
Never	3,847 (67.8)	39,967 (69.5)	2,253 (65.6)	30,669 (69.0)
Former	908 (16.0)	6,078 (10.6)	683 (19.9)	6,464 (14.6)
Current	916 (16.2)	11,413 (19.9)	499 (14.5)	7,301 (16.4)
Moderate activity ± SD, hours/week	0.9 (2.6)	0.9 (2.6)	0.9 (2.6)	0.9 (2.6)
Vigorous work ± SD, hours/week	0.3 (2.1)	0.6 (3.2)	0.6 (3.5)	0.6 (3.3)
Strenuous sports ± SD, hours/week	0.1 (0.9)	0.2 (1.0)	0.2 (0.9)	0.2 (1.0)
Coronary heart disease, n (%)	698 (12.3)	1,890 (3.3)	406 (11.8)	1,969 (4.4)
Stroke, n (%)	272 (4.8)	670 (1.2)	252 (7.3)	1,156 (2.6)

**Table 2:** Self-report prevalent diabetes at recruitment in relation to hazard ratio (HR) of total knee replacement (TKR) (n = 63,129).

	N	Cases	Person-years	Model 1	Model 2	Model 3
				HR (95% CI)	HR (95% CI)	HR (95% CI)
Total at recruitment						
No diabetes	57,458	1,857	843,748	1.00	1.00	1.00
Diabetes	5,671	116	69,016	0.70 (0.58–0.84)	0.63 (0.52–0.76)	0.63 (0.52–0.75)
Men at recruitment						
No diabetes	25,516	309	363,600	1.00	1.00	1.00
Diabetes	2,422	19	28,083	0.79 (0.50–1.26)	0.65 (0.41–1.03)	0.64 (0.40–1.02)
Women at recruitment						
No diabetes	31,942	1,548	480,148	1.00	1.00	1.00
Diabetes	3,249	97	40,934	0.68 (0.55–0.83)	0.62 (0.51–0.76)	0.63 (0.51–0.77)

HRs were adjusted for the following variables in separate models:

Model 1: age at recruitment (years); year of recruitment (1993–1995, 1995–1998); dialect group (Hokkien, Cantonese), level of education (no formal education, primary school, secondary school or higher).

Model 2: above adding BMI (model 2).

Model 3: model 2 adding self-reported histories of physician-diagnosed coronary heart disease or stroke, smoking status (never, former, or current), numbers of hours per week spent in moderate physical activity, vigorous work, and strenuous sports.

**Table 3:** Self-report incident diabetes at follow-up in relation to hazard ratio (HR) of total knee replacement (TKR) (n = 47,869).

	N	Cases	Person-years	Model 1	Model 2	Model 3
				HR (95% CI)	HR (95% CI)	HR (95% CI)
Total at follow-up						
No diabetes	44,434	1,276	434,427	1.00	1.00	1.00
Incident diabetes	3,435	89	31,713	0.94 (0.76–1.17)	0.74 (0.59–0.92)	0.75 (0.60–0.93)
Men at follow-up						
No diabetes	18,996	223	181,104	1.00	1.00	1.00
Incident diabetes	1,545	18	14,016	1.06 (0.65–1.71)	0.80 (0.49–1.30)	0.83 (0.51–1.34)
Women at follow-up						
No diabetes	25,438	1,053	253,323	1.00	1.00	1.00
Incident diabetes	1,890	71	17,698	0.91 (0.72–1.16)	0.72 (0.57–0.92)	0.74 (0.58–0.94)

HRs were adjusted for the following variables in separate models:

Model 1: age at follow-up (years); year of recruitment (1993–1995, 1995–1998); dialect group (Hokkien, Cantonese), level of education (no formal education, primary school, secondary school or higher).

Model 2: above adding BMI at follow-up (model 2).

Model 3: model 2 adding self-reported histories of physician-diagnosed coronary heart disease or stroke at follow-up, smoking status at follow-up (never, former, or current), numbers of hours per week spent in moderate physical activity, vigorous work, and strenuous sports.

**Table 4:** Self-report prevalent diabetes status at recruitment in relation to risk of total knee replacement stratified by body mass index (BMI) level.

	N	Case	Person-years	HR^a^ (95% CI)
BMI <23 kg/m^2^				
No diabetes	28,289	439	415,275	1.00
Diabetes	2,056	18	24,266	0.56 (0.35–0.90)
BMI 23 to <27.5 kg/m^2^				
No diabetes	24,499	1,023	361,312	1.00
Diabetes	2,857	73	35,172	0.66 (0.52–0.84)
BMI ≥27.5 kg/m^2^				
No diabetes	4,670	395	67,160	1.00
Diabetes	758	25	9,578	0.45 (0.30–0.67)

^a^HRs were adjusted for age at recruitment (years); year of recruitment (1993–1995, 1995–1998); dialect group (Hokkien, Cantonese), level of education (no formal education, primary school, secondary school or higher), BMI (kg/m^2^), self-reported histories of physician-diagnosed coronary heart disease or stroke, smoking status (never, former, or current), numbers of hours per week spent in moderate physical activity, vigorous work, and strenuous sports.
